# Mental health outcomes, literacy and service provision in low- and middle-income settings: a systematic review of the Democratic Republic of the Congo

**DOI:** 10.1038/s44184-023-00051-w

**Published:** 2024-03-06

**Authors:** Kayonda Hubert Ngamaba, Laddy Sedzo Lombo, Israël Kenda Makopa, Martin Webber, Jack M. Liuta, Joule Ntwan Madinga, Samuel Ma Miezi Mampunza, Cheyann Heap

**Affiliations:** 1 International Centre for Mental Health Social Research, Social Policy and Social Work, School for Business and Society, University of York, Heslington, York, YO10 5DD UK; 2 Centre Spécialisé dans la Prise en charge Psychosociale en Santé Mentale (CSPEMRDC), Université Chrétienne de Kinshasa, Kinshasa, Democratic Republic of Congo; 3 Neuropsychiatre et Addictologue Centre Spécialisé dans la Prise en charge Psychosociale en Santé Mentale (CSPEMRDC), Université Chrétienne de Kinshasa, Kinshasa, Democratic Republic of Congo; 4 Department of Health Sciences, University of York, Heslington, York, YO10 5DD UK; 5 WHO Country Office DRC & Medical Parasitology and Epidemiology, Faculty of Medicine, University of Kikwit, Kikwit, Democratic Republic of Congo; 6 Faculte de Medicine University of Kinshasa & Université Protestante au Congo (UPC), Kinshasa, Democratic Republic of Congo

**Keywords:** Psychology, Diseases, Health care, Signs and symptoms, Social sciences

## Abstract

In the Democratic Republic of the Congo (DRC), the prevalence of mental health issues could be greater than in other low-income and middle-income countries because of major risk factors related to armed conflicts and poverty. Given that mental health is an essential component of health, it is surprising that no systematic evaluation of mental health in the DRC has yet been undertaken. This study aims to undertake the first systematic review of mental health literacy and service provision in the DRC, to bridge this gap and inform those who need to develop an evidence base. This could support policymakers in tackling the issues related to limited mental health systems and service provision in DRC. Following Cochrane and PRISMA guidelines, a systematic (Web of Science, Medline, Public Health, PsycINFO, and Google Scholar) search was conducted (January 2000 and August 2023). Combinations of key blocks of terms were used in the search such as DRC, war zone, mental health, post-traumatic stress disorder (PTSD), anxiety, depression, sexual violence, war trauma, resilience, mental health systems and service provision. We followed additional sources from reference lists of included studies. Screening was completed in two stages: title and abstract search, and full-text screening for relevance and quality. Overall, 50 studies were included in the review; the majority of studies (*n* = 31) were conducted in the Eastern region of the DRC, a region devastated by war and sexual violence. Different instruments were used to measure participants’ mental health such as the Hopkins Symptoms Checklist (HSCL-25), The Harvard Trauma Questionnaire, Patient Health Questionnaire (PHQ-9); General Anxiety Disorder (GAD-7), and Positive and Negative Symptoms Scale (PANSS). Our study found that wartime sexual violence and extreme poverty are highly traumatic, and cause multiple, long-term mental health difficulties. We found that depression, anxiety, and PTSD were the most common problems in the DRC. Psychosocial interventions such as group therapy, family support, and socio-economic support were effective in reducing anxiety, depression, and PTSD symptoms. This systematic review calls attention to the need to support sexual violence survivors and many other Congolese people affected by traumatic events. This review also highlights the need for validating culturally appropriate measures, and the need for well-designed controlled intervention studies in low-income settings such as the DRC. Better public mental health systems and service provision could help to improve community cohesion, human resilience, and mental wellbeing. There is also an urgent need to address wider social issues such as poverty, stigma, and gender inequality in the DRC.

## Introduction

Poor mental health in low- and middle-income countries (LMICs) has become a real concern, due to its impact on human wellbeing, national disease burden, premature death, economic loss, and social cohesion^1,2^. Mental health is an integral component of health, defined as a state of physical, mental and social well-being and not merely the absence of disease or infirmity. According to the World Health Organization (WHO), mental health is “a state of well-being in which the individual realizes his or her own abilities, can cope with the normal stresses of life, can work productively and fruitfully, and is able to make a contribution to his or her community”^3^. Mental health conditions are problems involving changes in emotion, thinking or behaviour (or a combination of these), which are associated with distress and/or difficulties functioning in social, work or family activities^3^. Mental health is one of the most neglected areas of public health. Across the globe, close to one billion people are living with a diagnosis of mental disorder, and every 40 s one person dies by suicide^2^. Things have worsened in recent years as billions of people around the world have been affected by the COVID-19 pandemic^4,5^.

While many developed nations are making progress in supporting people with mental health conditions, in LMICs, more than 75% of people with mental, neurological and substance use problems receive no treatment or support at all. Unfortunately, stigma, discrimination, punitive legislation, lack of adequate health information, poor political will and human rights abuses are still widespread^1^. Additionally, a medical diagnostic model is the primary global mode of identifying mental health problems. The dominance of this approach and the limits of its biological treatments (such as drugs and hospital admission) are an additional threat to human rights. There is a real need to develop effective, especially psychosocial, mental health interventions in low-resource settings such as the DRC^1,2^. We therefore decided to undertake the first systematic review of the literature to examine the mental health literacy, symptoms, systems and service provision in DRC.

DRC is the largest country in sub-Saharan Africa, and because of its huge natural wealth and poor governance, DRC has suffered several wars including 1998 war involving nine African countries which was the deadliest conflict worldwide since World War II. Some authors describe DRC as in a chronic emergency, with endemic poverty, conflict, violence, forced dislocation of ethnic groups, and the use of torture and rape as weapons of war^6^, which have devastating effects on people’s mental health^7,8^. Previous studies have reported that people living in ‘humanitarian settings’ in LMICs such as DRC are exposed to a constellation of physical and psychological stressors that make them vulnerable to developing what are often called ‘mental disorders’^9^. On top of DRC’s war, the COVID-19 pandemic has affected health infrastructure^10^ and worsened the mental health problems of the population^11^. While many low-income countries have made some progress, the WHO 2019 report shows that DRC was not among 70 countries and territories that have so far prioritized coverage of mental health disorders^2^. This literature search aims to bridge this gap and inform those who need to develop an evidence base. We hope to help policymakers in tackling the issues related to limited mental health systems and service provision in DRC.

### Aims of the study

This study is the first systematic evaluation of mental health in low-resource settings of the DRC. The systematic evaluation looks at mental health literacy, symptoms, outcome measures, mental health systems and service provision in DRC. Mental health literacy has been defined as knowledge and beliefs about mental health disorders that aid their recognition, management, or prevention^12^. Mental health systems and service provision focused on DRC’s institutions and services that provide support to people with mental health conditions. The service provision included community-based support, respite for families and caregivers, traditional healers, and basic necessities such as shelter and clothing for people with mental health disorders^1,13^.

## Methods

The systematic review was conducted and reported according to Preferred Reporting Items for Systematic Reviews, Meta-Analyses (PRISMA), Cochrane Handbook recommendations^14,15^ and the COSMIN Risk of Bias checklist for systematic reviews^16^.

### Search strategy and data sources

**Fig. 1:**
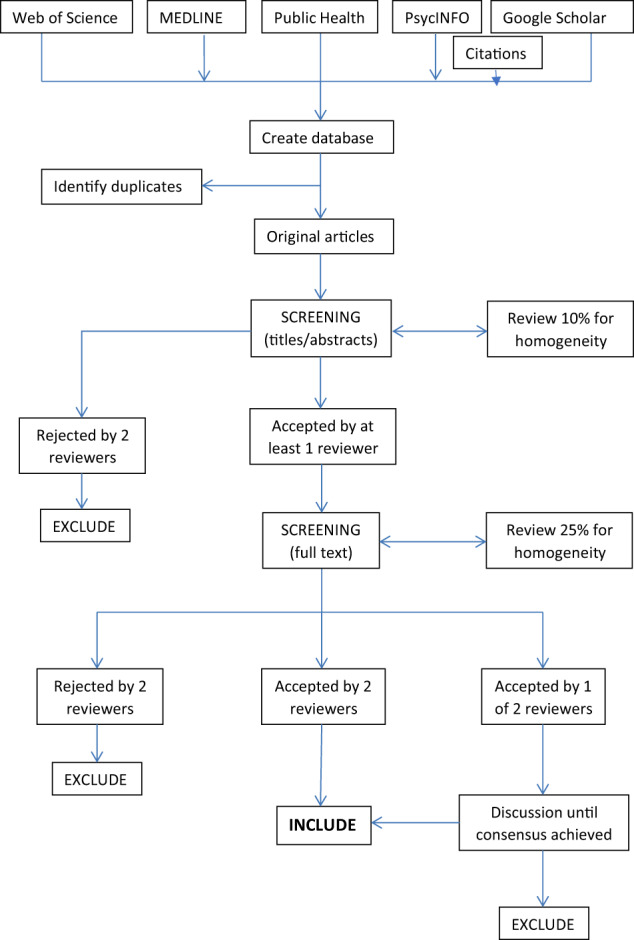
Search strategy and databases. The search strategy used in each of the databases.

Systematic searches of the literature published between January 2000 and August 2023 were carried out using Web of Science, MEDLINE, Public Health, PsycINFO and Google Scholar. Combinations of two key blocks of terms were used: (1) Democratic Republic of Congo, DRC, Zaire, Low-income country, low-income settings, Poor nations, Sub-Saharan country, War zone and (2) mental health, symptoms, outcome measures, validity assessment, PTSD, anxiety, depression, schizophrenia, psychosis, psychotic, ICD-10, rape, sexual violence, war trauma, mental health integration into general health care, and mental health systems and service provision. We also checked the reference lists of the studies meeting our inclusion criteria. Our search strategy used Jorm’s definition and conceptual framework to identify eligible studies^12^. The search strategy in each of the databases is presented in Supplementary Fig. 1. The search and screening process was conducted by two reviewers (Fig. 1).

### Study selection

**Fig. 2:**
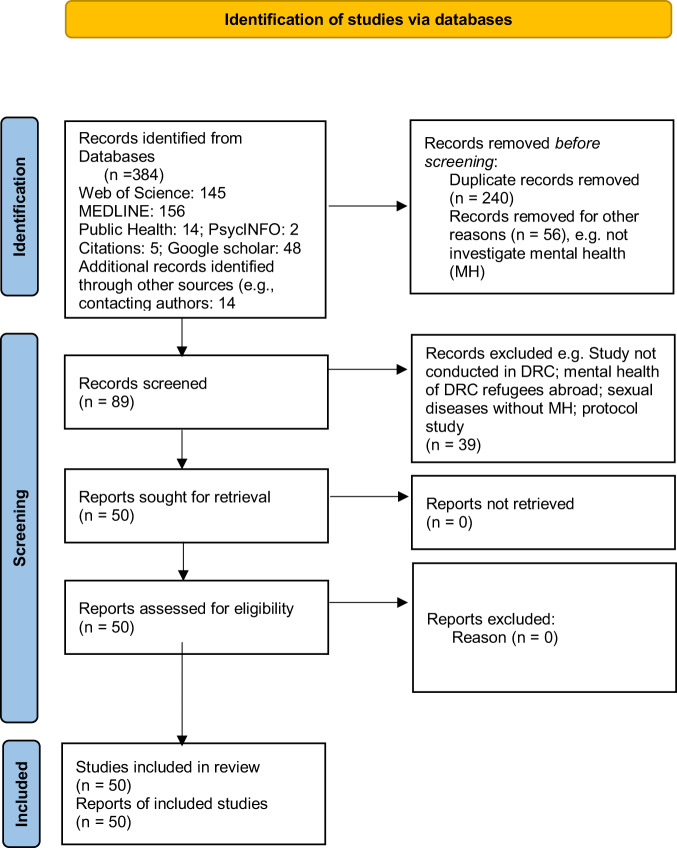
PRISMA flow diagram. The PRISMA flow diagram presents the screening process and selection of studies used in this systematic review.

Screening was completed in two stages. Initially, the titles and abstracts of the identified studies were screened for eligibility. Next, the full texts of studies initially assessed as “relevant” for the review were retrieved and checked against our inclusion/exclusion criteria. The screening process is presented in PRISMA Flow Diagram (Fig. 2).

### Eligibility criteria

Studies were eligible for inclusion if they met the following criteria: studies that have been conducted in DRC, and studies that have evaluated mental health literacy. Also included were the studies that assessed mental health service provision. Papers published in English and French were included, regardless of study design (e.g., qualitative, quantitative, randomized controlled trials, nonrandomized, descriptive studies, mixed-methods, and cluster randomized controlled trials). This systematic review therefore included studies that explored at least one of the main components of mental health literacy and/or service provision, which are: assessment of mental health, receiving a diagnostic label, understanding signs of poor mental health, training and health professionals, treatments, community-based support, prevention, stigma, abuses, and mental health institutions and management.

### Data extraction

An Excel file was devised for the purpose of data extraction. Two people conducted the data extraction and screening. This extraction was piloted across five randomly selected studies and changes were made where necessary to ensure inter-author consistency. Information about the following characteristics of the studies were extracted: first author’s name and year of publication, region/setting and sample, objective and research design, mental health outcome measure(s), findings, quality rating score, and comments/limitations. Another author confirmed the data extracted from each included study. Any discrepancy in the data obtained was discussed until a consensus was obtained.

### Quality appraisal and assessment

The quality appraisal was used to (a) find the most relevant studies, (b) get rid of irrelevant and weak studies, (c) separate evidence from opinions, and (d) identify any risk of bias. Following PRISMA and COSMIN recommendations, studies were rated for their quality by one researcher and verified by another researcher using criteria adapted from guidance on the quality assessment tools for quantitative studies^14,16,17^. Any disagreements were resolved by discussion. The quality review included assessment of (1) adequate information on population and recruitment methods, (2) robust research design, verified if (3) the mental health outcome measure used was valid and reliable, and determined if the (4) outcome variable was clearly identified and appropriate. The quality rating score was calculated by awarding one point for each of the criteria achieved (maximum 4). This appraisal process was done during the data extraction and verified after the systematic review was written.

## Results

We retrieved 384 studies. After removing duplicates (*n* = 240), studies were assessed and 56 articles were excluded after reading the titles and the abstracts for not investigating mental health disorders. Eighty-nine full-text studies were assessed and 39 articles were excluded for several reasons such as not using participants who were in the DRC; some studies looked at the mental health of refugees who were settling in other countries; and protocol studies were also excluded. Overall, 50 studies were included in the final analysis. While the quality appraisal was carefully and systematically followed, 19 of 50 studies were cross-sectional and investigated the association between key variables as we have described below. The flowchart of the screening and selection process^15^ is shown in Fig. 2.

### Descriptive characteristics of the studies

Table 1 presents the main characteristics of the 50 studies included in the review. All studies were conducted in the DRC. Thirty-one studies (64%) were conducted in the Eastern region of the DRC, a region devastated by war and sexual violence^6^. Among the remaining 19 studies, two were cross-national looking at the association between key factors^18,19^, four looked at the service provision at the national level^20–22^, eleven were conducted in the capital city Kinshasa, one was conducted in Vanga health zone in Central-West region, and one study was conducted in Equateur in the North-West region, and one study conducted in the southeast. All studies were published between 2005 and 2022. Sample sizes varied from 12 to 3941, with an average *M* = 543.2 (SD = 688.1). Participants were from different demographic categories including children and adolescents affected by war, children with epilepsy, female sexual violence survivors, survivors of Intimate Partner Violence (IPV), war-wounded men, people with psychosis, adults affected by the Ebola outbreak, postpartum mothers, psychiatrists, and members of organizations that support war-affected women and the general population. The majority of participants were people affected by war or women who had experienced sexual violence.

**Table 1:** Characteristics of included studies and quality ratings (MH = ‘mental health’).

No	1st Author & Year of publication	Region/setting Sample	Objective and research design	Mental health outcome measure(s)	Findings	Quality rating score	Comments
1.	Andersen, I. 2022^18^	DRC, Mali, Nigeria 2008 war-wounded DRC: 791 Mali: 538 Nigeria: 679	To identify patient characteristics associated with high distress prior to & after pre- and post-intervention. Mental health and psychosocial support (MHPSS) Logistic regression model, Cross-sectional	The 21-item Depression and Anxiety Scale (DASS21), the Impact of Events Scale Revised (IES-R) and the ICRC functionality scale before and after MHPSS intervention.	Following MHPSS, 92.28% of the patients showed an improvement on the DASS21, 93.00% showed an improvement on the IES-R and 83.04% showed an improvement on the ICRC Africa functioning scale.	4	Pre-test & follow-up Intervention: Psychosocial support (involvement of family and other caregivers in the MHPSS)
2.	Bass, J. 2016^23^	Eastern DRC 301 female sexual violence survivors.	Measures of economic and social functioning and mental health severity. Randomized controlled trial.	Economic and social functioning and mental health severity	Economic programme has a positive impact: Female sexual violence survivors with elevated mental health difficulties were successfully integrated into a community-based economic programme.	4	8-month follow-up
3.	Bass, J.K. 2013^36^	South & North Kivu province, DRC. 7 villages (therapy to 157 women) and 8 villages (individual support to 248 women)	To assess the impact of group therapy & individual support to Congolese survivors of sexual violence. Controlled trial	PTSD symptoms and combined depression and anxiety. Psychosocial functioning.	Group psychotherapy reduced PTSD scores, reduced combined depression and anxiety scores, and improved functioning.	4	Used robust assessment measures. Baseline and 6 months Follow-up.
4.	Bass, J. 2008^28^	Kinshasa, DRC. 133 women with and without the local depression syndrome.	To investigate post-partum depression syndrome among mothers in Kinshasa. Qualitative interviews.	Validating two standard depression measures: the Edinburgh Post-partum Depression Scale and the Hopkins Symptom Checklist.	Found a local syndrome that closely approximates the Western model of major depressive disorder. Useful for cross-cultural applicability and validation of the adapted screening instruments.	4.	
5.	Cenat J. M. 2022^34^	Equateur, DRC. 1614 adults affected by the ninth month of Ebola outbreak	Investigate the prevalence of, and risk factors associated with, depressive symptoms among individuals affected by Ebola Virus Disease (EVD) Multivariable logistic regression	EVD exposure level, stigmatization related to EVD and Beck Depression Inventory-Short Form (BDI-SF)	Adults in the two higher score categories of exposure to EVD were at two times higher risk of developing severe depressive symptoms.	4	
6.	Cherewick, M. 2016^35^	Eastern DRC. 434 male and female youth (aged 10–15 years)	Examine coping strategies among conflict-affected youth exposed to potentially traumatic events and the relationship to psychological symptoms and well-being. Hierarchical regression.	Measures of exposure to potentially traumatic events, an adapted coping strategies checklist, and measures of psychosocial distress and well-being.	Coping flexibility, or the use of multiple coping strategies, may be particularly useful in improving mental health and well-being.	3	
7.	Cikuru, J. 2021^24^	South Kivu, DRC. 167 women aged 15–69 years	Impact of music therapy group on women’s mental health. Step-wedged design, two pre-tests, a post-test, 3 & 6 months FU	Hopkins Symptoms Checklist (anxiety and depression). The Harvard Trauma Questionnaire (PTSD).	Significant improvement in women’s mental health: anxiety, depression, and PTSD 6 months after the intervention compared to baseline.	4	Intervention: Music therapy 3 & 6 months FU
8.	Corley, A. 2021^31^	South Kivu, DRC. 784 participants from 10 rural villages in South Kivu.	Investigate the association between attitudes towards gender equality, intimate partner violence (IPV) and mental health. Pearson’s chi-square test and logistic regression.Cross-sectional design.	Attitudes towards gender equality; IPV experiences; Hopkins Symptom Checklist-25 (HSCL-25) for anxiety and depression; Harvard Trauma Questionnaire (HTQ) for PTSD	Individuals in the moderately gender-equitable and fully gender-equitable classes had significantly lower mean scores on symptoms associated with PTSD than individuals in the least gender-equitable class.	4	Cross-sectional
9.	Dossa, N. I. 2015^61^	Goma, DRC. 320 women	To investigate the mental health disorders among women victims of sexual violence (SV). A cross-sectional design. Multivariate analyses	Post-traumatic stress disorder (PTSD) symptoms severity and psychological distress symptoms (PDS) severity	Experience of any SV was associated with more severe PDS. Only conflict-related sexual violence (CRSV) was associated with more severe PTSD symptoms.	3	Cross-sectional study
10.	Emerson, J.A. 2020^62^	South Kivu, DRC. 828 mothers of young children.	To investigate the association between mental health symptoms, and diet and nutritional status of mothers of young children. Cross-sectional design. Bivariate and multivariate regression analyses.	HSCL-25 and PTSD with the HTQ.	Mental health measures for women of young children were associated with higher dietary diversity scores. Mental health symptoms were not associated with body mass index.	3	Cross-sectional
11.	Espinoza, S. 2016^21^	DRC	Evaluating the Barriers to Mental Health Treatment within the Congolese Population. Descriptive case study	Risk factors to poor mental health include exposure to war, torture, and refugee camps. 39.7% of women and 23.6% of men have been exposed to sexual violence during their lifetime. 40.5% meet the criteria for major depressive disorder and 50.1% for PTSD after a 1-year recall period.	Barriers to MH: Different Perceptions of Mental Illness, Dependence on Treatment within their own Community, Lack of Mental Health Screening. Possible Interventions: Provide Service within the Community, CBT individual therapy and group therapy, and Education. Prevalence of sexual violence is higher.	3	Service provision
12.	Glass, N. 2017^37^	Eastern DRC. 833 household participants in 10 villages.	Test the effectiveness of livestock asset transfer intervention (Pigs for Peace) on mental health. Randomized controlled trial. From baseline to 18 months between the intervention and delayed control groups.	Harvard Trauma Questionnaire (HTQ) for PTSD; Hopkins Symptom Checklist (HSCL) for anxiety and depression; and Intimate partner violence (IPV).	The intervention increased economic stability, improved subjective health and mental health	4	18 months FU
13.	Glass, N. 2018^7^	Eastern DRC 188 adolescents and parents.	Parental and adolescent mental health and experience of intimate partner violence (IPV). Secondary analysis.	Parent PTSD and depression, subjected to IPV, Adolescent behaviours, stigma, and school attendance.	Parent mental health and IPV can have a negative impact on children’s well-being	4	8-month follow-up assessment
14.	Glass, N. 2012^38^	Eastern DRC. 50 women.	Case study of Congolese-US community-academic research partnership, to make an intervention to rebuild the lives of rape survivors and their families. Qualitative interviews	Poverty and traumatic stress for survivors. physical and mental health impact, stigma, exile; food security, employment; local availability of health care services and schools.	Survivors and family members experience significant health consequences of sexual violence. The survivor needs a way to regain her ‘worth’ in the family and the village. This study supports the feasibility of the international partnership.	4	
15.	Gerstl, S. 2011^46^	Eastern DRC. 552 randomly selected households	To determine the socio-economic conditions of the population and to assess their ability to contribute to health care. Service provision. Questionnaire cross-sectional	Evaluating the affordability of health services; fees and drug prices and whether free health care is possible.	Living conditions were very basic. Major source of income was agriculture (57%); 47% of the households earned less than US$5.5/week. 92% able to contribute to consultation fees (max $0.27) and 79% to the drug prices (max $1.10). 6% opted for free consultations and 19% for free drugs.	4	Service provision and affordability
16.	Ikanga, J. 2014^53^	DRC. General population	Psychology in the DRC; Service provision	Evaluating the contribution of psychological departments to improve MH conditions. Evaluating access to Mental health facilities	Mental health facilities lacking psychological departments in the DRC need to be known. Partnership is needed between Western psychology and Congolese culture.	3	Service provision
17.	Johnson, K. 2010^8^	Eastern DRC 998 households.	Explore the link between sexual violence and human rights violations, and physical and mental health. Cross-sectional study Structured interviews and questionnaires.	Measures sexual violence prevalence, symptoms of major depressive disorder (MDD) and PTSD, human rights abuses, and physical and mental health needs.	Self-reported sexual violence and other human rights violations were prevalent and were associated with poorer physical and mental health outcomes. 41% (*n* = 374/991) met the criteria for MDD and 50.1% met the criteria for PTSD.	3	
18.	Kangoy. A. K. 2016^39^	Eastern DRC. 69 adults.	To investigate the mental health consequences of rape for the survivor. Questionnaires Cross-sectional	Post Traumatic Syndrome Disorder (PTSD), Major Depressive Disorder (MDD), comorbid PTSD/depression	Social rejection, the characteristic of the rape event and the residential area were significantly related to the severity of mental health consequences for the survivor.	3	
19.	Kashala, E. 2005^63^	Kinshasa 1187 children, 7–9 years old	To investigate mental health problems, and the association between these problems and school performance, demographic factors, illness and nutrition. Questionnaire cross-sectional	Mental health problems were assessed with the Strengths and Difficulties Questionnaire (SDQ), a questionnaire on child behaviours administered to teachers.	Poor nutrition, low socioeconomic status and illness increased the risk for mental health problems and low school performance.	3	
20.	Kitoko, G. M. B. 2019^33^	Kinshasa 60: 30 patients with schizophrenia & 30 healthy participants	Identify possible deficits in facial emotion recognition among patients with schizophrenia. Descriptive and correlations	Diagnosed with schizophrenia according to DSM-5 criteria Beck depression inventory; positive and negative symptoms scale (PANSS)	Patients with schizophrenia had emotion recognition deficits, particularly for negative emotions	3	Cross-sectional
21.	Kohli, A. 2014^64^	Eastern DRC. 315 women in 10 villages.	Relationship among conflict-related trauma, family rejection, and mental health in adult women living in rural eastern DRC. Questionnaires and interviews.	Exposure to trauma, sexual assault, family rejection, and mental health (PTSD and depression).	Exposure to conflict-related trauma, including sexual assault, was associated with an increased likelihood of family rejection, and poorer mental health outcomes.	4	
22.	Koegler, E. 2019^65^	Eastern DRC 12 members of solidarity groups for female survivors of sexual violence.	Exploring the impact of joining the solidarity group and factors that contributed to the mental health of female survivors of conflict-related sexual violence. Interviews.	Physiological, psychological, economic, or social measures	All women identified some improvement (physiological, psychological, economic, or social) since joining the solidarity group, but none of the women were free from personal distress.	3	Qualitative data
23.	Koegler, E. 2018^66^	Eastern DRC. 753 adults	Association between mental health and sexually transmitted infections (STIs) in conflict-affected settings Regression analysis	Depression, anxiety, PTSD and STIs	People with higher scores on mental health measures were more likely to be treated for an STI than those with lower scores.	4	
24.	Kohli, A. 2015^67^	Eastern DRC. 701 women	Association between trauma experiences, PTSD, depression and amount of social interaction. Regression analysis	Trauma experiences, PTSD, depression and the amount of social interaction	Increased trauma was associated with fewer visitors to women’s homes, and fewer visits to the homes of family/community members.	4	
25.	Kohli, A. 2012^68^	Eastern DRC. 772 women survivors of sexual violence in 6 rural villages	Case study focused on: 1. expansion of mobile clinic services; 2. evaluation system; and 3. brief psychosocial support Case study: descriptive	Anxiety, depression, PTSD, social dysfunction, suicide ideation.	85% of participants reported being survivors of sexual violence; 45% never received health services after the last sexual assault. Participants experienced anxiety (29.8%), sadness (43.8%), and shame (34.4%).	3	Case study descriptive
26.	Lieberman Lawry, L. 2022^69^	Beni, Butembo and Katwa health zones in DRC. 223 adult Ebola survivors, 102 sexual partners & 74 comparison respondents.	To understand the prevalence of mental health problems in Ebola-affected communities, and their association with condom use. Case study	Post-traumatic stress disorder (PTSD), depression, anxiety, substance use, suicidal ideation and attempts, stigma, and sexual behaviour.	Survivors met symptom criteria for depression at higher rates than partners. PTSD symptom criteria for survivors were four times greater than the comparison participants.	3	Cross-sectional
27.	Lokuge, K. 2014^19^	DRC, Iraq and the occupied Palestinian Territory (oPt). 3025 individuals, 20 years of age. DRC (14%), Iraq (17,5%) and oPt (51%).	Evaluating Mental health services for children exposed to armed conflict. Consultation Brief trauma-focused therapy, the current MSF mental health therapeutic intervention. Descriptive cross-sectional	Anxiety-related,mood-related, behaviour-related and somatisation problems.	Brief trauma-focused therapy, the current MSF mental health therapeutic intervention, appears to be effective in reducing symptoms. 45.7% left programmes early.	4	
28.	Ngamaba, H. K. 2022^32^	Kinshasa, DRC 100 individuals, general population	Quality of life (MANSA), prevalence of depression & anxiety during COVID-19.	MANSA, EQ-5D-3L, UCLA, PHQ-9, GAD-7.	Depression and anxiety are more prevalent. Negative link between MANSA and living alone.	4	
29.	Ngoma, M. 2010^70^	Kinshasa, DRC 341: 153 healthy control subjects vs 188 patients	Cognitive deficits in nonaffective functional psychoses	Cognitive assessment, PANSS, Antipsychotic drug	Patients perform significantly worse than healthy controls on all cognitive domains with cognitive deficits being most pronounced in verbal and working memory, attention, motor speed, and executive function	4	
30.	Mankuta, D. 2012^43^	Eastern DRC 441 women- sexual trauma victims	To test an intervention programme: training local staff; medical evaluation and treatment of patients; psychological treatment of trauma victims. Intervention case study	PTSD and the psychological treatment based on EMDR (eye movement desensitization and reprocessing) principles.	Training local staff showed improved knowledge, enhance awareness and providing them with tools to diagnose and treat sexual assault and mutilation.	4	Intervention: Training staff
31.	Masika, Y. D. 2019^71^	Eastern DRC 302 participants	Influences of trauma awareness and preparedness on the development of PTSD. ANOVA, Relationships, Mediation	Posttraumatic Checklist Scale, General Self-Efficacy Scale, and Traumatic Events List	Trauma awareness and preparedness play an important role among military personnel in moderating the risk of developing PTSD, more so than among the civilian population	4	
32.	Masika, Y. D. 2019^72^	Eastern DRC 120 individuals	Association between peritraumatic dissociation (PD) and PTSD in individuals exposed to recurrent armed conflict. Descriptive cross-sectional	Traumatic Events List, the Peritraumatic Dissociative Experiences Questionnaire, and the French version of the PTSD Checklist Scale	The group of participants with high scores for PD had significantly more PTSD. The primary target population for prevention and early management should comprise individuals with high levels of PD, low levels of education, and women.	3	Cross-sectional
33.	Matonda-Ma-Nzuzi, 2018^73^	Kinshasa DRC 104 children with epilepsy (CWE)	Factors associated with behavioural problems and cognitive impairment in CWE Descriptive and Multivariate analysis	The Child Behaviour Checklist (CBCL); the Wechsler Nonverbal (WNV) scale of ability	Behavioural problems and cognitive impairment are common in CWE. Behavioural problems were associated with socioeconomic features only	3	
34.	Mukala, Mayolo E. 2023^41^	Lubumbashi, DRC 591 residents responded and conducted 5 focus groups with 50 key stakeholders (doctors, nurses, managers, community health workers. and leaders, health care users)	Integrating mental health care into the primary care system. Survey and Focus groups	Evaluating the integration of mental health care into the primary care system in one region	The burden of mental health problems is a major public problem in Lubumbashi. The outpatient curative consultations are low at 5.3%. There are no dedicated psychiatric beds, nor is there a psychiatrist or psychologist available		Service provision Cross-sectional
35.	Mukala, Mayoyo E. 2021^42^	Lubero District Eastern DRC 3941 used the services offered	To investigate the integration of a mental health care package into the general health care system. Case study design	Evaluating the Integration of mental health care package into the general health care System. 7 new cases/1000 inhabitants/year	3941 patients with mental health problems used the care offered at the health centers and the district hospital between 2012 and 2015. It is possible to integrate mental health into existing general health services in the DRC.	4	Integration of mental health
36.	Mels, C. 2010^29^	Ituri district in Eastern DRC 1046 adolescents (13–21 years) in 13 secondary schools.	Validating two broadly used mental health self-report measures--Impact of Event Scale-Revised (IES-R) and Hopkins Symptom Checklist 37 for Adolescents (HSCL-37A). Focus groups and interviews	Self-report measures—Impact of Event Scale-Revised (IES-R) and Hopkins Symptom Checklist 37 for Adolescents (HSCL-37A).	Community-based adaptation can extend the validity of the measures. The availability of adequate Swahili and Congolese French adaptations of the IES-R and HSCL-37A could stimulate the assessment of psychosocial needs in DRC	3	Validating measures
37.	Mudji, J. 2022^74^	Vanga health zone in Kwilu in Bandundu. 93 patients	Investigate mental distress and health-related quality of life in people with gambiense human African trypanosomiasis. *T*-test and chi2 or Fisher’s exact tests. Structured interviews	Hospital Anxiety and Depression Scale, Becks Depression Inventory and the 36-item Short Form Health.	The presence of neurological sequelae leads to mental distress and a diminished QoL. Depression and anxiety were higher in former patients with neurologic sequelae. The QoL scores were lower.	4	Structured interviews
38.	Mukongo K. J. 2019^47^	Kinshasa 136 caregivers working at the CNPP	The contribution of caregivers and holistic support of people with MH problems. Descriptive and correlation. Observation, interview and questionnaires.	Outpatient support Evaluating the work of caregivers giving Holistic support to persons with mental disorders.	Caregivers are needed to support people with MH conditions. They need transport to visit patients. 53.8% of careers were between the ages of 41–60; 69.8% were males. 69.8% were nurses (2nd level) and 34.6% had 31–40 years experience.	3	Outpatient support for 6 months plus
39.	Ndjukendi, A, 2017^30^	Kinshasa Zone de santé de Masina II 66 adolescents	Adolescents experiencing difficulties in Kinshasa: what coping strategies are used? semi-structured two-phase evaluation Cross-sectional study	Temperament according to Eysenck, parenting style according to Baumrind, maternal attachment interview adapted for adolescents, and coping strategies according to Spirito’s Kidcope.	Support for adolescents experiencing difficulties should focus on strengthening socialization functions and adaptive resources.	3	Coping strategies
40.	O’Callaghan, P. 2014^25^	North-eastern DRC 159 war-affected children and young people	Investigate the outcome of support for war-exposed youth at risk of attack and abduction. 8 sessions of a group-based, community-participative, psychosocial intervention. Pre- and post-intervention.	Symptoms of post-traumatic stress reactions, internalizing problems, conduct problems and pro-social behaviour.	At post-test, participants reported significantly fewer symptoms of post-traumatic stress reactions compared to controls. At 3-month follow-up, moderate to large improvements.	4	Intervention: group-based psychosocial Pre- and post-intervention and at 3-month follow-up
41.	On’okoko, M. O. 2010^20^	DRC National level	Map existing service provision and evaluate the delivery of mental healthcare. Service provision Descriptive case study	Map existing service provision and evaluate the outcomes of services: Mental health policy and legislation. Mental disorders. Psychiatric services. Mental health workforce.	Mental health policy and legislation exist but no government budget. Popular beliefs persist about supernatural causes. Mental disorders are as common as they are elsewhere, but there is no national epidemiological data. 6–15% of schizophrenia; 22% of anxiety disorders; 13–23% of mood disorders.	4	Service provision
42.	OSAR, 2022^40^	DRC	[Access to psychiatric health care] accès à des soins psychiatriques service provision	Availability and limited capacity of mental health care; High costs of mental health care; Drug availability and costs	Fewer than 60 neuropsychiatrists in the whole country; Six MH hospitals; people with mental disorders can receive care in secondary and tertiary institutions; Lack of qualified personnel; High costs Psychiatric daily rates, clinical admission: $10–20, Inpatient treatment Public $20–25, Private $50, Specialist consultation Psychiatrist $15–25, Psychiatrist nurse $10; Psychologist $10, CBT $10, EMDR $25. Stigma: often considered “cursed”, no possibility of recovery.	4	Service provision
43.	Schalinski, I. 2011^75^	Eastern RDC 53 female survivors of war	Examine relationships between the number of traumatizing events, degree of shutdown dissociation, PTSD, and depression. Cross-sectional study, A path-analytic model	PTSD, and depression.	Cumulative exposure and dissociation were associated with increased PTSD severity. PTSD and witnessing predicted depression. PTSD mediated the link between dissociation and depression.	4	
44.	Schuster, A. 2013^27^	Kinshasa (the capital)	Map existing service provision Secondary analysis	Psychiatric treatment, Stigma, Informal support, Training need	Lack of Psychiatric treatment, Stigma affecting informal support, Lack of MH professional training Lack of MH services.	3	
45.	Scott, J. 2015^76^	Bukavu, DRC 757 adult women raising children from sexual violence-related pregnancies (SVRPs). Cross-sectional Descriptive analysis.	Assess mental health outcomes among women raising children from SVRPs, and stigma toward and acceptance of women and their children. Cross-sectional	Patient Health Questionnaire PHQ-9, GAD-7, the PTSD Checklist-Civilian Version (PCL-C), and Suicidal ideation and attempt, and Perceived stigma.	48.6% met symptom criteria for major depressive disorder, 57.9% for post-traumatic stress disorder, 43.3% for anxiety and 34.2% reported suicidality. Women who reported stigma were more likely to meet symptom criteria.	4	
46.	Taylor, S. 2017^45^	Kinshasa, DRC interviews with 16 psychiatrists	Develop a greater understanding of mental health interventions to diminish the treatment gap in Kinshasa Interviews	Mental health interventions an alternative epistemological framework is needed.	There is a need to increase the global availability of mental health services. Critical treatment practices: thinking with and beyond biomedicine.	3	Service delivery
47.	Vaillant, J. 2023^77^	Eastern DRC 1053 women	Link between mental health disorders (PTSD, depression and/or anxiety) and employment for women in conflict zone. RCT of Narrative Exposure Therapy (NET)	PTSD, depression and/or anxiety.	A positive relationship between work or working hours and increased symptoms of PTSD and depression and/or anxiety. Working women with worse PTSD and depression and/or anxiety symptoms are also less likely to be self-employed.	4	RCT baseline & FU
48.	Verelst, A. 2014^78^	Bunia, eastern Congo 1305 school-going adolescent girls aged 11–23	Investigate the link between sexual violence and mental health of eastern Congolese adolescents and its differing associations with daily stressors, stigma, and the labelling of sexual violence Questionnaire Cross-sectional	Self-report measures of mental health symptoms, war-related traumatic events, experiences of sexual violence, daily stressors, and stigmatization were administered	Daily stressors, stigmatization, and war-related events showed a large impact on girls’ mental health. Link between sexual violence (rape or non-consensual sexual experiences) and poorer mental health.	3	Cross-sectional
49.	Vivalya, B. M. 2020^44^	North-Kivu Province, DRC	Implementing of mental health services in an area affected by prolonged war and Ebola disease outbreak. Case study service provision	Mental health services The deficiencies of mental health services in North-Kivu and solutions on how to provide holistic mental health services in the presence of an ongoing war and highly contagious epidemic.	There are deficiencies of mental health services and no functional work plan is in place. The need for integrative training programmes, Advocacy and social mobilization, Provision of emergency MH services, and Com. outreach.	3	Service Provision
50.	Wachter, K. 2018^26^	Eastern DRC 744 women who experienced sexual violence.	Investigate the relationship between social support, internalized and perceived stigma, and mental health. Secondary cross-sectional regression analysis	Social support variables, felt stigma, and depression, anxiety and PTSD.	Emotional support seeking and felt stigma were positively associated with increased symptom severity of depression, anxiety and PTSD.	4	

HSCL-25: The Hopkins Symptom Checklist-25; HTQ: Harvard Trauma Questionnaire; PTSD: Post-Traumatic Stress Disorder; PHQ-9: Patient Health Questionnaire; GAD-7: General Anxiety Disorder. We follow PRISMA and COSMIN recommendations and the quality rating score was calculated by awarding 1 point for each of the criteria: (1) population and recruitment methods, (2) research design, (3) if mental health outcome measure was valid and reliable and (4) if outcome variable was clearly identified.

Most studies collected primary data using questionnaires, interviews, and observation. Six studies included longitudinal follow-ups^23–25^. Few studies used secondary data to investigate women who experienced sexual violence in Eastern DRC^26^, and service provision and psychiatric treatment in Kinshasa^27^. Several designs were used including descriptive, correlational, causal-comparative/quasi-experimental, and experimental research. Randomized controlled trials and step-wedged design were used where participants were pre-tested and post-tested 3 and 6 months later^24^, and 8 months later^23^. Eighteen studies were cross-sectional and descriptive. Three studies used qualitative ethnographic and case study designs where participants were interviewed with semi-structured interviews and focus groups^28–30^.

### Mental health outcome measures and validity assessment

Different instruments were used to measure participants’ mental health (e.g. anxiety, depression, and PTSD), partner intimate violence, stigma, experience of sexual violence, and exposure to adversity (e.g. Ebola virus outbreaks). Most studies assessed depression (*n* = 19), anxiety (n = 15), and PTSD (*n* = 14) symptoms of their participants. For anxiety and depression, the Hopkins Symptoms Checklist (HSCL-25) was the most common measure^24,31^. One recent study used both Patient Health Questionnaire (PHQ-9) and General Anxiety Disorder (GAD-7) to assess the prevalence of depression and anxiety during the pandemic COVID-19^32^. The Harvard Trauma Questionnaire was the most common measure used to assess PTSD. Positive and negative symptoms scale (PANSS), for example, was used to identify possible deficits in facial emotion recognition among patients with schizophrenia^33^. Stressful life events were assessed using the Impact of Event Scale revised version (IES-R)^29^. To assess stigma, a 20-item scale that measures 20 possible forms of stigmatization related to Ebola Virus Disease (EVD) was used^34^. Most instrument measures used multiple items to assess participants’ mental health and other conditions. Of 50 studies, 21 studies used questionnaires adapted from other standardized measures used in other countries. One study validated two broadly used mental health self-report measures: the Impact of Event Scale Revised (IES-R) and the Hopkins Symptom Checklist 37 for Adolescents (HSCL-37A)^29^, and another study validated two standard depression measures: the Edinburgh Post-partum Depression Scale and the Hopkins Symptom Checklist^28^. A small number of two studies used self-designed surveys to measure the positive impact of socio-economic projects on the mental health and well-being of sexual violence survivors^23^, or young people in war zones^35^.

### Psychosocial interventions

Six studies looked at psychosocial interventions, where four focussed on group therapy and family support, and the other two investigated socio-economic support.

Amongst the studies that focussed on group and family therapy, we noticed variation in the way participants were supported. For example, after 6-month follow-ups, group psychotherapy reduced PTSD symptoms and combined depression and anxiety symptoms among Congolese women survivors of sexual violence^36^. A cross-national study conducted in DRC, Mali and Nigeria found that the involvement of family and other caregivers in psychosocial support reduced the symptoms of depression and anxiety among war-wounded men^18^. Similar positive findings were found in another cross-national study, where brief trauma-focused therapy and Medicine Sans Frontier (MSF) mental health therapeutic intervention were used among young people. Brief trauma-focused therapy appears to be effective in reducing symptoms among young people exposed to armed conflict in DRC, Iraq and the Occupied Palestinian Territory^19^. Moreover, a 12-week music session and community engagement programme led by a psychologist and music producer were associated with significant improvement in women’s mental health, which was sustained up to 6 months post-completion of the programme, despite instability in the region and evidence of continued experience of conflict-related trauma^24^.

Regarding socio-economic support, two studies reported that people living in war-affected zones of the DRC are often poor and have limited access to traditional financial institutions. However, microfinance programmes have the potential to help in improving income, economic productivity and mental health^37^. Two studies found that group-based economic interventions were effective to support female sexual violence survivors^23,37^. An innovative productive asset transfer programme, Pigs for Peace (PFP), increased economic stability, improved subjective health and mental health in 10 conflict-affected villages^37^.

### Stigma and rejection

Five studies highlighted the stigma and rejection. The stigma around mental health issues and social rejection were depicted in various ways across the reviewed studies. For example, a study on sexual violence survivors revealed that rape survivors need a way to regain their “worth” in the family and the village^38^. Many women experienced significant physical and mental health consequences of sexual violence and were rejected because of the stigma around mental health and the violence itself^39^. The social rejection was closely linked with spousal rejection. The perceived loss of dignity, the shame of living with a woman who had experienced rape, and the influence of the family members were contributing factors to spousal rejection^39^. However, gender-based violence is not a mental health problem: contributors to violence against women include social norms and attitudes, economic inequality, and women’s lack of socio-political power. Mental health support should sit alongside social and structural interventions such as economic help^23^ and addressing attitudes that enable violence against women^31^.

Our search suggested that mental health awareness may help to reduce some general stigma around mental health difficulties, because many people in the DRC region believe that mental health problems are a curse of witchcraft, or caused by bad spirits^40^. Social stigma and rejection can link to local beliefs about mental distress: a family may prefer to go to a traditional healer, or to an exorcist pastor/priest to pray rather than seek more ‘professional’ interventions^40^. This study found that some people may believe that the consequences of the war are only physical, and ignore the consequences of the war from a psychological point of view^20^.

### Mental health systems, service provision and training

There are very few hospitals for the treatment of mental health disorders in DRC. The country has only six public psychiatric hospitals, and a dozen private mental health centres with 500 beds for nearly 90 million inhabitants, almost all of which are in big cities^41,42^.

Among the few well-known specialized mental health facilities, Kinshasa, the capital city, has two mental health hospitals, the Centre Neuro-Psycho Psychiatrique de Kinshasa (CNPP) run by the University of Kinshasa, and the Telema Mental Health Centre which is managed by the Roman Catholic Church. In provinces, DRC has: the CNPP at the Katwambi Centre (Centre de Katwambi) in the province of Western Kasai; the Doctor Joseph Guillain of Lubumbashi Neuropsychiatric Centre (Centre Neuropsychiatrique Docteur Joseph Guillain de Lubumbashi); the Department of Neuropsychiatry of Sendwe Hospital in Lubumbashi in Katanga province; and the psychiatric facilities in the South-Kivu province called Centre Psychiatrique de Soins de Santé Mentale (SOSAME) in Bukavu^20,41^. We also note how this lack of hospital provision links to the need for a change of focus towards the social causes of poor mental health. It’s unlikely that existing mental health training has yet caught up with this mandate for community-centred and social (rather than biological) treatments, even in those few existing hospitals.

Three studies in this review highlighted the need for training local staff^20,43,44^. An intervention programme for 441 women sexual trauma victims found that training local staff showed improved knowledge, enhanced awareness and provided them with tools to recognize sexual assault and to provide psychological support^43^. Another study, implementing mental health services in an area affected by prolonged war and Ebola disease outbreak, found deficiencies in mental health services, and no functional work plan was in place. However, integrative training programmes, advocacy and social mobilization, provision of emergency mental health services, and community outreaches were needed in the region^44^.

DRC’s mental health policy was formulated in 1999 but so far, there are no budgetary allocations for mental health. The DRC mental health policy promotes a recovery approach to mental health care, which emphasizes support for individuals to achieve their aspirations and goals. Unfortunately, not much has been done due to the lack of a budget allocated to mental health^20,40,41^.

### Integration of mental health care into the general health care and who can afford health cost

A study conducted in the eastern DRC, looking at the experience of integrating mental health care into the general health care system, found that it is possible. 3941 patients used care offered at health centres and the district hospital between 2012 and 2015, and an average of 7 new cases/1000 inhabitants per year was recorded^42^. Moreover, a study that interviewed 16 psychiatrists in Kinshasa supported the idea that mental health care can be integrated into general health care if new ways of approaching global mental health are applied. For example, using more responsive forms of support which acknowledge the value of patient experiences^45^, and are not limited to the reductive rationalism typical of the biological paradigm^45^. A household survey to which 591 residents responded and five focus group discussions (FGDs) were held with 50 key stakeholders (doctors, nurses, managers, community health workers and leaders, health care users) found that the integration of mental health care into the primary care system is difficult in Lubumbashi due to the lack of service provision^41^. For example, the study found that there are no dedicated psychiatric beds, nor is there a psychiatrist or psychologist available. Participants in the FGDs stated that in this context, the main source of care for people remains traditional medicine^41^.

Another study looking at who can afford health care found that most of the Congolese population struggles to afford health care costs because 47% of households earn <US $5.50/week^46^. Figures suggest that diagnosable mental health disorders are as common in the DRC as elsewhere: 6–15% of people meet the criteria for schizophrenia; 22% for anxiety disorders; and 13–23% for mood disorders^20^. Yet, individuals and their families absorb costs related to drugs, treatment, food, bedding, and hospitalization in a country where most people live on less than US$2 per day^46,47^. The impact of this financial burden is greater for women, as they have less income^48^. Interviews with 552 households found that to afford health care people may sacrifice other basic needs such as food and education, with serious consequences for the household or individuals within it. However, 92% said that they were able to contribute to treatment consultation fees (max. $0.27) and 79% were able to pay for any drug prices (max. $1.10); 6% opted for free consultations and 19% for free drugs^46^. This demonstrates again the need for community-based treatments that use and bolster existing community resources, rather than relying on hospital stays that families can ill afford.

Mental health care as it stands is expensive, and costs for professional support vary from public to private. The daily rates for public psychiatric treatment are US$10–20 for outpatients, or US$20–25 for inpatients; private inpatient treatment costs double (US$50). A specialist consultation with a psychiatrist costs $15–25; Eye Movement Desensitization and Reprocessing (EMDR) is US$25; and other professionals cost US$10 (psychiatric nurse, psychologist, or a session of Cognitive Behavioural Therapy (CBT))^40^. It is worth noting that in a country with a significant number of people traumatized by war and sexual violence, trauma-based therapy (EMDR) is the most expensive treatment.

## Discussion

This systematic review highlighted a clear demand for mental health care. The prevalence of mental health issues is greatly increased by major risk factors related to armed conflicts and poverty. The review covered the whole DRC with a particular focus on the eastern part of the country. Mental health problems are under investigated in the DRC. The number of studies found is small and not consistent with the extent and significance of mental health problems caused by war-related sexual violence. DRC in general, and the eastern region in particular, has been devastated by war and sexual violence. Many voices have been raised to condemn the atrocities, including Nobel Peace Prize winner Dr Denis Mukwege, who has called for an end to the use of rape as a weapon of war^49^. In line with previous work, our study found that wartime sexual violence and extreme poverty are highly traumatic, and cause multiple, long-term mental health difficulties^6,50^. We found that depression, anxiety, and PTSD were the most common problems in the DRC. Similarly, other systematic reviews in conflict-affected populations find high frequencies of mental health illnesses such as depression, anxiety, post‐traumatic stress disorder, bipolar disorder, and schizophrenia^51^, and PTSD among civilians who have experienced sexual violence^50^.

This review found that existing mental health services in the DRC are limited. ‘Mental health’ diagnosis may sit in opposition to local beliefs, leading to a lack of uptake in existing services. People with mental health illnesses in DRC and many other sub-Saharan African countries are more likely to seek help from traditional healers and religious leaders^52–54^. Hence, there needs to be collaboration with local communities and a pluralistic framework of understanding^45^. Some problems identified in this review, such as stigma and rejection, sit within the social realm. Positive social connections are important for physical and mental wellbeing. They can provide emotional support, practical assistance, information and a sense of belonging^55^. However, ‘social support’ is not always positive^26^, hence it’s crucial to understand a person’s needs within their local context. Additionally, more non-medical mental health interventions are required—for example in the current review, help with livestock had a positive impact on mental health^37^.

To address the mental health treatment gap in LMICs, then, there is a need to develop psychosocial interventions that are culturally appropriate and embedded in local knowledge, values and practices^56^. Although most medical and psychological interventions have been developed and evaluated in high-income countries, this review found positive effects for psycho-social interventions such as group therapy, music therapy, family support, and socio-economic projects^18,36,37^. This matches previous research in humanitarian settings, which supports the efficacy of psychosocial interventions for adults with common mental disorders^57^, and therapy for reducing suicidal ideation^58^. Still, applying these findings to poor-resource settings might be a challenge^57^, and in the DRC there is a lack of related health professionals from social work, psychology, and occupational therapy^40^. Effectively measuring the outcomes of such interventions will also be crucial in building the evidence base. Yet, whilst this review found common standardized measures in the literature (e.g. Hopkins Symptom Checklist), only two studies tried to validate these Western measures in the DRC context. This included the Impact of Event Scale-Revised (IES-R), two variations of the Hopkins Symptom Checklist, and the Edinburgh Post-partum Depression Scale^28,29^. As such, further studies are required to ensure that measurements are both valid and reliable for the DRC context.

Finally, this review highlighted a lack of mental health institutions, and the need to train more mental health professionals to tackle stigma, reduce social rejection and provide support^20,38,39,59^. We highlight the need for a greater breadth of professionals (including social work, psychology, and occupational therapy), and acknowledge that institutions are not the best or only way to support mental health. As such, more research is needed into social and community mental health interventions in the DRC. Moreover, this review highlights the need to integrate mental health care into general health care. Existing mental health care is unaffordable for many Congolese people^46^. Unfortunately, there are no governmental budget allocations for mental health, and there is no national epidemiological data on mental health^20,59^.

As such, despite the global impact of mental health disorders, mental health service provision in LMICs (and the DRC specifically) is inadequate. Previous studies have urged for the prioritization of mental health services in budgets and service planning, with an emphasis on incorporating local population and cultural needs. One study in this review found that training local staff improved their ability to support survivors of sexual violence^43^. Another study highlights that mental health provision in LMICs can be achieved only from a foundation of political will and strengthened legislation, including resource allocation, strategic organization, integrated care provided by sufficiently trained staff, and the meaningful involvement of patients, informal carers, and the wider community^1^. In the DRC, political will is still required to back up policy and legislation with funding. Addressing the research gaps noted above will strengthen the argument for improved mental health services, and provide evidence-based solutions to mental health needs in the DRC.

### Limitations

First, the methodological weakness is that many studies (e.g. 19 of 50) were cross-sectional and descriptive. Second, the current search identified studies from predominantly eastern DRC (64% of included studies) which, limits the generalizability of findings to other regions of the DRC. The large number of studies in the eastern region of the DRC is however justified because of war, pervasive sexual violence, and generally poorer mental health^60^. Third, this systematic review did not conduct a meta-analysis because of the lack of appropriate data. Hence, the findings are presented narratively.

## Conclusion

This systematic review calls attention to the need to support sexual violence survivors and many other Congolese people affected by traumatic events. This review also highlights the need for validating culturally appropriate measures and the need for well-designed controlled intervention studies in the DRC. Better public mental health systems and service provision could help to improve community cohesion, resilience, mental well-being, and even economic productivity. There is also an urgent need to address wider social issues such as poverty, stigma, and gender inequality in the DRC. More evidence is needed on reducing mental health stigma in the DRC. Further collaboration with communities is required to ensure people are willing and able to access available services.

## Supplementary information

Supplementary information

## Data Availability

The authors confirm that the data supporting the findings of this study are available within the article [and/or] its supplementary materials.
