# Supplementary figures and images for: Mental health outcomes, literacy and service provision in low- and middle-income settings: a systematic review of the Democratic Republic of the Congo
Source: Npj Ment Health Res. 2024 Mar 6;3:9. doi: 10.1038/s44184-023-00051-w (PMC10956021; doi:10.1038/s44184-023-00051-w)

Supplementary Figure 1. Search strategy and databases.

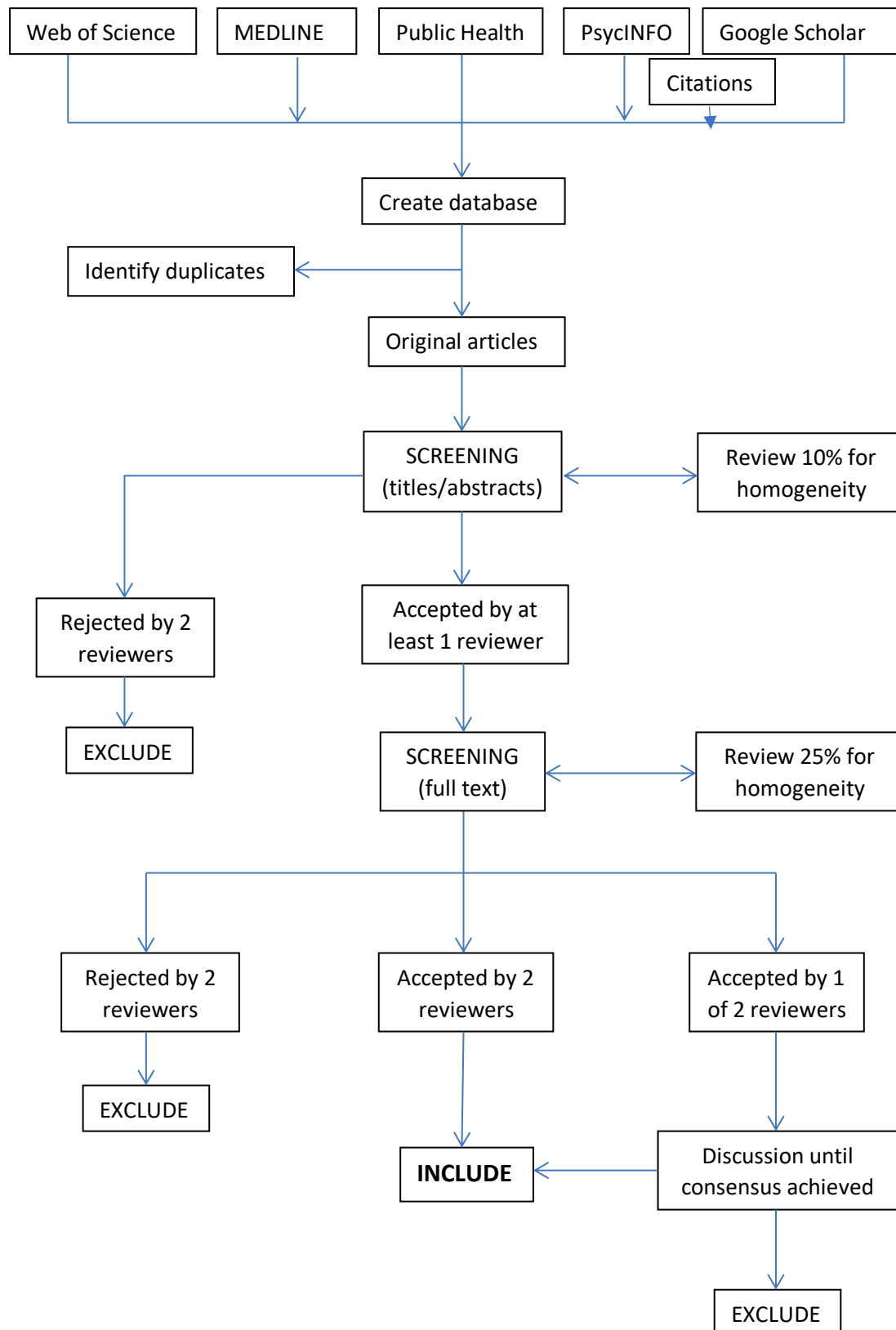

Supplement: Supplementary file 1 — Supplementary information [file 44184_2023_51_MOESM1_ESM.pdf]
